# Epidemiology to public health intervention for preventing cardiovascular diseases: The role of translational research

**DOI:** 10.4103/0971-5916.73421

**Published:** 2010-11

**Authors:** Anand Krishnan, Kapil Yadav, Manmeet Kaur, Rajesh Kumar

**Affiliations:** Centre for Community Medicine, All India Institute of Medical Sciences New Delhi India; * *School of Public Health, Postgraduate Institute of Medical Education & Research, Chandigarh, India*

**Keywords:** Cardiovascular diseases, public health interventions, translational research

## Abstract

Despite significant progress in medical research, cardiovascular diseases (CVDs) continue to be the largest contributors of morbidity and mortality both in developed and developing countries. The status of public health interventions related to CVDs prevention was reviewed to identify actions that are required to bridge the existing gap between the evidence and the policy. We used a framework comprising two steps - “bench to bedside” and from “bedside to community” to evaluate translational research. Available literature was reviewed to document the current status of CVD prevention and control at national level in India. Case studies of risk factor surveillance, tobacco control and blood pressure measurement were used to understand different aspects of translational research. National level initiatives in non-communicable diseases surveillance, prevention and control are a recent phenomena in India. The delay in translation of research to policy has occurred primarily at the second level, *i.e*., from ‘bedside to community’. The possible reasons for this were: inappropriate perception of the problem by policy makers and programme managers, lack of global public health guidelines and tools, and inadequate nationally relevant research related to operationalization and cost of public health interventions. Public health fraternity, both nationally and internationally, needs to establish institutional mechanisms to strengthen human resource capacity to initiate and monitor the process of translational research in India. Larger public interest demands that focus should shift to overcoming the barriers at community level translation. Only this will ensure that the extraordinary scientific advances of this century are rapidly translated for the benefit of more than one billion Indians.

## Introduction

Translational research has been characterized as “effective translation of the new knowledge, mechanisms and techniques generated by advances in basic science research into new approaches for prevention, diagnosis, and treatment of diseases which is essential for improving health”^1^. A translational researcher has been defined as “someone who takes something from basic research to a patient and measures an endpoint in a patient.”^2^. Traditionally translational research has been defined in a narrow sense, *i.e*., from ‘laboratory discovery to clinical use’ or “Bench to Bedside” (T1)^3^. However, in recent times, a distinction has been made into a second area of translational research that seeks to close treatment gaps and improve quality by improving access, reorganizing and coordinating systems of care (T2)^4^.

In order to understand the process of translational research, a framework is presented in the Table that builds on the available frameworks^5^,^6^. There are broadly three “As” in this framework.

**Table:** Conceptual Framework to understand phases in translational research

Steps		Players involved	Conceptual end point	Type of activity/research required	Source of funding
Awareness	Knowledge acquisition	Basic science researchers in laboratory	First reported study	Basic innovative science research	Pharmaceutical & biotechnology firms
	Knowledge Validation	Basic/clinical researcher	Other studies confirming the finding (at least 5??)	Basic science Research	
Acceptability	Knowledge transfer	Clinicians	Enters clinical practice	Clinical research including trials	
	Knowledge dissemination	Professional bodies	Enters standard clinical management guidelines	Meta-analysis & consensus development	Professional bodies and academic sector
Application	Knowledge Application	Public health researchers	Development of public health protocols/guidelines	Operational research & consensus development	Donor agencies/governments
	Knowledge translation	Governments	Included in a goVernment funded national programme	Economic analysis and advocacy	Donor agencies/governments

As modified from Refs^5^,^6^

*Awareness*: This phase relates to the actual invention (knowledge acquisition) or discovery and its validation by other researchers before it is accepted as an evidence. The actors involved in this phase are basic science researchers and main funding for this phase comes from pharmaceutical or biotechnology companies. Government funded initiatives are also present in many developed countries.

*Acceptability*: Second phase starts when the discovery is put to practical use in clinical settings. While the initiation would be by individual clinicians, professional bodies constitute expert groups to develop or modify the guidelines by using the techniques of meta-analysis and consensus development. Clinical trials are largely funded by pharmaceutical agencies and the guidelines development is generally led by professional bodies, often funded indirectly by pharmaceutical and biotechnology firms.

*Application*: Third phase of translational research starts when an accepted clinical practice tries to find its way into a national level policy, plan or programme. It has to enter into the public health research agenda since health service or operational research would be needed to test its feasibility for a large-scale programme. Appropriate economic analyses and advocacy are required in this phase to convince the policy makers about its cost-effectiveness. Multilateral development agencies and/or governments generally fund this effort.

As a corollary of the above mentioned three phases of translational research, there are two major interfaces. The first interface is of “Bench to Bedside” or between the basic researcher and the clinician (T1), and the second interface is between “Bedside to Community”, *i.e*., between the clinician and the public health researcher (T2). These interfaces are critical for the transfer of evidence from one phase to the other, finally resulting into a successful translation of research to public policy/programme.

In this paper, we have defined the end point of translational activity as national policy, plan or programme, therefore, our main focus is on translation of research into national programme *i.e*. T2. This is especially true for countries like India, where public sector is still a major channel for delivery of affordable health services to the poor especially in rural areas. We use the frameworks mentioned above to describe the current status of translating evidence into policy or programme with regards to prevention of cardiovascular diseases (CVDs) in India. We analyze three case studies and use them to identify the gaps and the reasons for delay in translating evidence to policy or programme with regards to CVDs. This understanding could then lead to identification of translational activities and research to strengthen such efforts in general and with specific reference to cardiovascular diseases.

## Status of evidence on CVDs

*Burden of disease*: CVDs, which were responsible for less than 10 per cent of all deaths at the beginning of last century, now cause about 30 per cent of the deaths globally^7^. In India, CVDs cause about 27 per cent of the deaths, indicating faster pace of epidemiological transition. In the last two decades, several epidemiological studies have documented the rising burden of CVDs^8^,^9^.

*Evidence on causal pathways*: The causal pathways of CVDs and the concept of risk factors have been well identified globally since the Framingham Study^10^. Studies among South Asians have discovered some unique features of these diseases such as early onset, increased severity, and higher risk and role of obesity and metabolic syndrome^11^–^14^.

*Availability and affordability of effective interventions*: CVD prevention and control policies and programmes require involvement of several sectors to control consumption of tobacco, alcohol, salt, sugar and fat, and to promote use of fruits and vegetables and physical activity. These strategies have been well articulated globally^15^,^16^. The cost of population-wide policy and educational interventions for reducing salt intake from current level by 15 per cent and reduction in tobacco control in 28 developing countries has been estimated as 0.40 US$ per person per year. The cost of multi-drug regimen delivery to high risk individuals for reducing the risk of cardiovascular diseases was estimated as 1.08 US$ per person per year. Thus about US$ 1.5 per person per year is required in the national budget of low income and lower middle income countries for launching policy and programmes that have a potential to achieve a reduction of 2 per cent per year in the NCDs^9^,^17^.

## Situational analysis of public health interventions for CVD prevention in india

Until recently, very little of the epidemiological research on CVDs has been translated into public health practice in India. The essential public health functions of CVD programme would include components of surveillance, prevention and control. Current status of these three components is discussed below:

CVD surveillance occurs at three levels – mortality, morbidity and risk factors. Surveillance of causes of death has only been strengthened recently in India^18^. Though there have been ad-hoc disease surveys, a systematic approach to tracking diseases does not exist, probably due to the complexities involved in such a venture. Globally, also, the focus of surveillance has been on risk factor surveillance.

India ratified The Framework Convention on Tobacco Control (FCTC) on February 5, 2004 and a Tobacco Control Legislation has been enacted^19^. Changes in the Prevention of Food Adulteration Act, to make the labelling of the quantity and type of nutrient in processed/packaged food, are underway to provide healthier choices to consumers. The National Programme for Prevention and Control of Diabetes, CVD and Stroke, launched as a pilot programme on January 4, 2008, in nine districts, is expected to cover entire country in the near future^20^.

## Understanding the determinants of success gap in translation

Case studies of risk factor surveillance, blood pressure management, and tobacco control were analysed to understand the determinants of the success and gaps in the translation process (Box). It is concluded that at national level critical components that are missing are: standard disease management guidelines, diet and physical activity guidelines, mechanisms for sensitization of policy makers, and guidelines for involvement of non-health sectors. Unlike the success achieved in tobacco, alcohol control has not been successful, despite the fact that the issues are similar except that alcohol use is perceived to be more as a social issue than a health issue. Existence of persistent gaps in successful translation indicates the role of strong lobbies and industries in policy and programme formulation.

**Box**

**Translating evidence to programme - Case studies from cardiovascular diseases in India**

**Case study 1: CVD surveillance – case of risk factors**

Status: The role of risk factors in CVD has been well established since seventies. While monitoring of risk factor levels as a part of management has been a well established clinical practice, till recently it has not been a part of public health surveillance. Behavioural Risk Factor Surveillance (BRFS) has been in practice in US since 1984^21^. World Health Organization (WHO) developed the global tool of Steps approach only in 2003^22^. The Government of India initiated non-communicable disease (NCDs) risk factor surveillance as a part of the National Integrated Disease Surveillance Programme in 2007^23^. During the interim period, this protocol was adapted and pilot tested in six sites in India.

Lessons: The delay between evidence and programme has been primarily due to lack of availability of standard procedures, guidelines and tools. Two other contributory factors could be the lack of perceived need for addressing NCDs and less emphasis on surveillance in our health system. Once a standard global tool was evolved, the uptake of surveillance initiatives in India has been fast.

**Case study 2 : CVD control – case of hypertension measurement**

Status: In 1905, Korotkoff discovered a simple and precise technique to measure arterial pressure^24^. It took another 34 years for it to be recognized and officially accepted by American Heart Association and the Cardiac Society of Great Britain25. Currently, it is used extensively by clinicians, but its use in larger health system in the Indian context *i.e*. by para-health professionals is limited to that of inclusion as a package of antenatal care. The expansion of services for blood pressure measurement was hindered due to the need of use of stethoscope and the hesitation among medical fraternity to allow its use by para-health professionals. This has been circumvented now by the availability of valid digital blood pressure measuring instruments. WHO recently developed the protocol for management of CVDs in different setting, including those where physicians are not present and this has been tested for its effectiveness in India^26^–^28^.

Lessons: It shows that the translation of the discovery into clinical decision making was much faster but its use into a public health system globally and in India has been slow. The primary reason for the slow translation has been lack of appropriate technology and lack of simplified guidelines for assessment and management partly supported by inadequate communication of epidemiological evidence about the rising burden of the disease into the policy arena and resistance by clinicians to a public health approach to these diseases

**Case Study 3 : CVD prevention – case of tobacco**

Status: Initial epidemiological evidence associating smoking to lung cancer came from the classic study by Doll & Hill in 1950^29^. Over the next decade many studies confirmed this association. This led to the international acceptance of tobacco as cause of lung cancer between 1962–64^30^. A series of tobacco control measures including a ban on advertising were instituted in many of the developed countries. After strong advocacy efforts especially by non-governmental organizations, a significant anti-tobacco lobby emerged which succeeded in launching many initiatives for tobacco control, and the campaign culminated in the adoption of Framework Convention for Tobacco Control (FCTC) in 2003^16^. India was among the first few countries to ratify and sign the treaty. However, still there is limited information on effectiveness of different tobacco control measures.

Lessons: The probable reasons for successful translation were transformation of a medical issue into a social cause by strong involvement of NGOs, high political commitment, and good stewardship by WHO. In tobacco prevention, the clinical stage was largely bypassed.

One of the lessons learnt from the case studies (Box) is that a quick adaptation and uptake of practice occurs once globally validated tools or frameworks are made available. The primary reason for the delay has been the non-availability of global guidelines and tools. This indicates the failure of the way the global public health system functions. In contrast, the professional bodies (probably funded by pharmaceutical companies) have quickly translated research into clinical practice. Some of the other factors contributing to the delay have been the resistance from the clinical establishments to public health approach to these diseases, and misperceptions about the seriousness of the problem among policy makers and programme managers.

Many of the reasons for lack of translation to policy/programme highlighted above would apply to public health interventions for other diseases as well. In contrast, public health programmes like oral rehydration therapy (ORT) for management of diarrhoeal diseases and universal salt iodization (USI) for prevention of iodine deficiency disorders (IDD) have been effectively translated. The probable reasons for success in case of ORT were: perception of diarrhoea as a number one killer of children, simplicity of the intervention, and endorsement by professional bodies such as Indian Academy of Pediatrics and Indian Association of Preventive and Social Medicine. For IDD, key ingredients of success were: feasibility and effectiveness of salt iodization nationally, advocacy among key stakeholder at highest political level, and continuous support from multilateral agencies.

## How can we expedite the translation process?

Given that the need and evidence for CVD prevention exists along with gaps in the implementation, how do we influence the process of policy or programme development to hasten the process of bridging the gaps. In order to influence it, translational researchers need to understand the process of converting the evidence-based public health interventions into policies and programmes. Grindle & Thomas suggest that policy development occurs in two phase^31^. The first is the agenda setting phase when a particular issue is considered for inclusion in the policy agenda. The process of developing policy does not begin until policymakers are convinced that the issue is important enough for them to spend time on. The next phase is the actual formulation of the policy. Porter^32^ building on the work of Kingdon^33^, proposed that three different processes occur almost independently of each other (*i*) problems are identified and described; (*ii*) solutions that may address existing problems are proposed; and (*iii*) political openings to address the problems appear and disappear. In other words, the problems and solutions need to be linked.

Lavis *et al*^34^ developed a framework for assessing country-level efforts to link research to action. The framework has four elements: general climate, production of research, push/pull factors, and approach to evaluation. Nuyens & Lansang^35^ drew the following lessons from knowledge translation initiatives. First, the system context is paramount and all efforts have to be linked to the existing health systems. Second, continuity is important. Third, complexity of the health system should be considered and the efforts need to be adapted to the specific layer of the health system. Fourth, all stakeholders should be involved. And finally, capacities are the weakest link^35^. Let us look at these issues in our context.

*General climate*: Currently there is a high political commitment within the Ministry of Health & Family Welfare at Central government level^36^. While commitment at highest level can lead to development of a programme, it needs support of all the players from senior programme managers to the village level health workers for it to be successfully implemented and sustained.

*Presence of an institutional mechanism for debate and decision making*: This should be at both technical as well as policy level. It is important to have forums where informal and formal discussion with all the concerned stakeholders can be held. This mechanism would weigh different aspects of the policy and put it up for a final decision, which in the case of India would be the national government through its statutory bodies. Currently, issues are not debated adequately, either in the public or among scientific circles in India. The decisions are often ad-hoc as exemplified by the ban on sale of non-iodized salt for human consumption which was first banned, then revoked and then again banned^37^. There is an imminent need to put in place institutional mechanisms for policy development, implementation and monitoring in India.

*Push and pull factors*: To break the inertia of policy development, we need to have push or pull forces acting on the system. Pull factors are those which result in a creation of demand for the services or programme among the intended utilizers. This could be looked at as not only the general community but also the process by which one could make the policy makers and programme mangers “demand” a programme. The basic process here is awareness generation and advocacy efforts. Efforts on this front require making the complex evidence base that includes effectiveness, cost, acceptability, technological and organizational feasibility, *etc*. available to policy makers in a format that is understandable by them. There is also a need for mobilizing the community through health promotion efforts using mass media, systems like schools, workplaces, *etc*.

The push factors are generally in the form of a “champion/crusader” or other lobbies like industry, drug companies. In the absence of an institutional framework, some person or an agency plays the role of a policy “champion”. This member of the policy elite (researcher, politician, professional association, non-government organization) engages in continuous advocacy among peers (in related ministries and in civil society) and then shepherds the issue through the policy making process, once the political opening appears. Though the role of scientists and researchers as “champions” of a cause is debatable, in many developing countries including India researchers are usually the champions and advocates of policy changes^38^. With regards to NCD prevention and control, there are powerful lobbies in terms of diet, alcohol, tobacco, drinks and marketing which have the potential to influence the process of policy development. Counteracting such pressures or working with such stakeholders requires much expertise and brinkmanship which is currently lacking in the country.

*Linking clinicians and public health community*: There is a larger delay of translation of clinical trials to public health intervention. The faster acceptance in the first interface, *i.e*., laboratory to clinical practice is due to better funding, better interaction between the concerned groups and an immediate benefit for the users of the research - clinicians. This is not true for the second interface, *i.e*., between clinical and public health sector. The inter-link between clinicians and public health researchers is largely non-existent. The funding source, *i.e*., pharmaceutical companies span across the basic researcher-clinician interface but no such entity (public health council) exists that can extend this link to the second interface, *i.e*., clinician-public health researcher. Cardiovascular diseases are primarily seen as clinical disease entities and the upstream causes or “cause of causes” identified by the social determinants of health are not adequately addressed. There is only very little understanding of the public health approach to CVDs prevention among clinicians, policy makers and programme managers.

*Resource generation for policy development*: The responsibility for providing resources for the policy process is often spread across the interested parties. Government may provide funds to develop policy, while professionals and community groups provide expertise and skill to inform the policy process. The role of private sector in resource generation is important but one needs to keep in mind the conflicts of interest with respect to CVDs prevention which involves dealing with tobacco, alcohol and food industry. To moderate the role of external influences, it is necessary to create a transparent and effective institutional framework.

*From policy to programme*: It is true that many policies have been made but these have not been converted into a programme. Operationalization of the policy requires preparation of programme implementation plans including the mechanisms of monitoring and evaluation. Establishment of an integrated CVD programme is a complex task and operational research is needed to address many relevant issues. The public health fraternity in India has not considered this as a major challenge so far. A look at the recent issues of the public health journals from India, as observed by authors shows that studies on operational research are very few. This is an area requiring urgent attention by the public health fraternity and by the agencies that fund such research activities in India. Health sector has always received less financial allocation than what is needed for provision of a basic package of services. Only in recent years has allocation to health sector been increased at national level but this is yet to happen at State level.

## Role of translational research(ers)

It is obvious that the question of why research is not being translated into programmes is itself a good area for research. As translation of epidemiological research into policy and practice involves a complex process, often additional research is required in social, economic, technological, administrative, and policy domains to pave the way for formulation of evidence-based policy and programmes. A translational researcher needs to understand how policies are made, what are the information needs of policymakers, how this information is to be made available, operational research to support programme planning and implementation, understanding advocacy strategies (what works and on whom), *etc*. It is clear that this is not a linear process as it was largely believed but a complex and dynamic process.

The role of translational research in different disciplines of medicine like cardiology, neurology or oncology is being increasingly debated, as researchers worldover are anguished by the paradox of major breakthroughs in biological research, be it in DNA or nanotechnology, co-existing with huge preventable disease burden in the world especially in the developing countries^39^–^41^. The scope of knowledge and expertise needed for an effective translational scientist cannot be acquired “on the job”. There is a need for training in wide range of skill sets that span biomedical, behavioural, operational, economical, and ethical sciences. National Institutes of Health (NIH), USA, has started a special scheme for translational scientists and is creating a new academic discipline^42^. The challenges encountered and the promise during the initial years of this federally funded national level health care initiative in United States has been recently published^43^. There is no denying the fact that there is an urgent need to create mechanisms to foster translation research that promote evidence-based public health policy and practice in India as well.

## Conclusion

There is enormous amount of evidence related to CVD prevention and control which needs to be brought to patients and communities, but progress in this direction is slow. Currently, translational research seems to be focusing more on first level (bench to bedside; T1) rather than the second level (bedside to community) in most countries including India. While both levels are important, each level faces different challenges. Considering the fact that many laboratory and clinical researches have still not been translated into public health interventions in India, larger public interest demands that focus should be on overcoming the barriers identified above. Understanding the process of translational research through which epidemiological research can find application into public systems can help catalyze the development of public health programmes to stem the rising tide of CVDs in India. This will ensure that the extraordinary scientific advances of this century are rapidly disseminated, assimilated and translated for the benefit of more than one billion Indians.

## References

[1] Fontanarosa PB, DeAngelis CD (2002). Basic science and translational research in JAMA. *JAMA*.

[2] Birmingham K (2002). What is translational research?. *Nat Med*.

[3] Kasbekar DP (2005). B2B-2K5: The new buzz in translational research. *Curr Sci*.

[4] Woolf SH (2008). The meaning of translational research and why it matters. *JAMA*.

[5] Alving B The road map ahead for clinical and translational science. http//nihroadmap.nih.gov/clinicalresearch/B10_2006_Alving.ppt.

[6] Zerhouni EA, Alving B (2006). Clinical and translational science awards: a framework for a national research agenda. *Transl Res*.

[7] Murray CJL, Lopez AD, Murray CJL, Lopez AD (1996). A comprehensive assessment of mortality and disability from diseases, injuries and risk factors in 1990 and projected to 2020. *The global burden of disease*.

[8] (2004). *Assessment of burden of non-communicable diseases*.

[9] (2005). NCMH Background Papers. *Burden of disease in India*.

[10] Kannel WB, Dawber TR, Kagan A, Revotskie N, Stokes J (1961). Factors of risk in the development of coronary heart disease--six year follow-up experience. The Framingham Study. *Ann Intern Med*.

[11] Jha P, Enas EA, Yusuf S (1993). Coronary artery disease in Asian Indian: prevalence and risk factors. *Asian Am Pac Isl J Health*.

[12] Enas EA, Metha J (1995). Malignant coronary artery disease in young Asian Indians: thoughts on pathogenesis, prevention, and therapy. Coronary Artery Disease in Asian Indian (CADI) Study. *Clin Cardiol*.

[13] Vallapuri S, Gupta D, Talwar KK, Billie M, Mehta MC, Morise AP (2002). Comparison of atherosclerotic risk factors in Asian Indian and American Caucasian patients with angiographic coronary artery disease. *Am J Cardiol*.

[14] Joshi P, Islam S, Pais P, Reddy KS, Dorairaj P, Kazmi K (2007). Risk factors for early myocardial infarction in South Asians compared with individuals in other countries. *JAMA*.

[15] Geneva, WHO. 2004. *Global strategy on diet, physical activity and health*.

[16] Geneva, WHO. 2005. *Framework convention on tobacco control*.

[17] Beaglehole R, Ebrahim S, Reddy S, Voûte J, Leeder S (2007). Chronic Disease Action Group. Prevention of chronic diseases: a call to action. *Lancet*.

[18] Jha P, Gajalakshmi V, Gupta PC, Kumar R, Mony P, Dhingra N (2006). RGI-CGHR Prospective Study Collaborators. Prospective study of one million deaths in India: rationale, design, and validation results. *PLoS Med*.

[19] Reddy KS, Gupta PC (2004). *Report on tobacco control in India*.

[20] (2008). Press Information Bureau. Government of India. *Pilot phase of the national programme for prevention and control of diabetes, cardiovascular diseases and stroke launched*.

[21] (2008). CDC. Scientific Data Documentation. *Behavioral risk factor surveillance systems (1989)*.

[22] (2008). WHO. Surveillance in brief. 2004 Nov.; Issue 8. http://wwwwhoint/ncd_surveillance/news/surb8_webpdf.

[23] (2008). Integrated Disease Surveillance Project. http://mohfwnicin/depth12htm.

[24] Korotkoff NS (1905). A contribution to the problem of methods for the determination of the blood pressure [in Russian]. *Bull Imperial Mil Med Acad*.

[25] Paskalev D, Kircheva A, Krivoshiev S (2005). A centenary of auscultatory blood pressure measurement: a tribute to Nikolai Korotkoff. *Kidney Blood Press Res*.

[26] Mendis S, Lindholm LH, Mancia G, Whitworth J, Alderman M, Lim S (2007). World Health Organization (WHO) and International Society of Hypertension (ISH) risk prediction charts: assessment of cardiovascular risk for prevention and control of cardiovascular disease in low and middle-income countries. *J Hypertens*.

[27] Abegunde DO, Shengelia B, Luyten A, Cameron A, Celletti F, Nishtar S (2007). Can non-physician health-care workers assess and manage cardiovascular risk in primary care?. *Bull World Health Organ*.

[28] Kar SS, Thakur JS, Jain S, Kumar R (2008). Cardiovascular disease risk management in a primary health care setting of north India. *Indian Heart J*.

[29] Doll R, Hill AB (1950). Smoking and carcinoma of the lung: preliminary report. *Br Med J*.

[30] (1964). US Department of Health, Education and Welfare. *Smoking and health*.

[31] Grindle MS, Thomas JW (1991). *Public choices and policy change: The political economy of reform in developing countries*.

[32] Porter RW (1995). *Knowledge utilization and the process of policy formulation: Toward a framework for Africa*.

[33] Kingdon JW (1984). *Agendas, alternatives, and public policies*.

[34] Lavis JN, Lomas J, Hamid M, Sewankambo NK (2006). Assessing country-level efforts to link research to action. *Bull World Health Organ*.

[35] Nuyens Y, Lansang MA (2006). Knowledge translation: linking the past to the future. *Bull World Health Organ*.

[36] Srinath Reddy K, Shah B, Varghese C, Ramadoss A (2005). Responding to the threat of chronic diseases in India. *Lancet*.

[37] Anand K, Baridalyne N, Moorthy D, Kapoor SK, Sankar R, Pandav CS (2002). Ethical issues in public health policy. *Natl Med J India*.

[38] Rothman KJ (1993). Policy recommendations in epidemiology research papers. *Epidemiology*.

[39] Finkelstein R, Miller T, Baughman R (2002). The challenge of translational research-a perspective from the NINDS. *Nat Neurosci*.

[40] Baumann M, Bentzen SM, Doerr W, Joiner MC, Saunders M, Tannock IF (2001). The translational research chain: is it delivering the goods?. *Int J Radiat Oncol Biol Phys*.

[41] Weinrauch L (2008). What is translational research in cardiology?. http://wwwhealthcentralcom/heart-disease/c/77/20111/research/.

[42] Zerhouni EA (2005). Translational and clinical science - time for a new vision. *N Engl J Med*.

[43] Califf RM, Berglund L (2010). Principal Investigators of National Institutes of Health Clinical and Translational Science Awards. Linking scientific discovery and better health for the nation: the first three years of the NIH’s Clinical and Translational Science Awards. *Acad Med*.

